# Knowledge, Attitudes, and Behaviors on Utilizing Mobile Health Technology for TB in Indonesia: A Qualitative Pilot Study

**DOI:** 10.3389/fpubh.2020.531514

**Published:** 2020-10-06

**Authors:** Dewi Nur Aisyah, Riris Andono Ahmad, Wayan Tunas Artama, Wiku Adisasmito, Haniena Diva, Andrew C. Hayward, Zisis Kozlakidis

**Affiliations:** ^1^Indonesia One Health University Network (INDOHUN), Depok, Indonesia; ^2^Infectious Disease Informatics, Institute of Health Informatics, University College London, London, United Kingdom; ^3^Center for Tropical Medicine, Gadjah Mada University, Yogyakarta, Indonesia; ^4^One Health Collaborating Center (OHCC), Faculty of Veterinary Medicine Gadjah Mada University, Yogyakarta, Indonesia; ^5^Faculty of Public Health, Universitas Indonesia, Depok, Indonesia; ^6^Institute of Epidemiology and Health Care, University College London, London, United Kingdom; ^7^International Agency for Research on Cancer, World Health Organization, Lyon, France

**Keywords:** tuberculosis, Indonesia, mobile health, community based research, qualitative study

## Abstract

Tuberculosis (TB) infections remain a global health burden with a high incidence rate in South-East Asia, including Indonesia. TB control strategy is founded on early case detection and complete treatment to minimize transmission and prevent the emergence of drug resistance. However, many patients face challenges to comply with daily medication, causing many to adhere inconsistently or stop prematurely. Technological solutions could enhance adherence to treatment and support national screening and follow-up policies. These include telephone video communication, enabling health professionals to watch patients take their medication, address patients' concerns, and provide advice and support. This manuscript describes the outcome of a qualitative pilot study, based on a series of focus group discussions to assess the knowledge, attitudes, and behaviors, on the potential utilization of mobile technology for health purposes with a particular focus on TB treatment follow-up. The findings illustrate that general knowledge of mobile health technologies, of their legal framework of operations, and of their exact potential within the healthcare system is incomplete or poor. The novel findings are as follows: (a) the willingness of participants to learn about these technologies, (b) the open and welcoming attitude toward receiving such information even within frontline community settings, and (c) the willingness to back a government-supported, healthcare-driven set of such initiatives. Potential implementation barriers have also been highlighted. This study is an important first step toward understanding the attitudes and behaviors on utilizing mobile health technology for TB in Indonesia.

## Introduction

Tuberculosis (TB) is one of the infectious diseases caused by *Mycobacterium tuberculosis* with millions of new infections reported globally and a significant number of deaths. In 2014, 1.4 million people globally died from TB, reducing slightly to 1.2 million in 2018; with an estimated 10 million new infections reported per year over the last few years (1, 2). TB affects people in both sexes of all age groups. It infects the lungs and other organs such as the glands, skin, bones, and brain. Thus, TB can potentially infect anyone but predominantly affects the poor. Globally, 98% of TB deaths are in the poorest countries (3), while most TB cases in 2018 were in the WHO regions of South-East Asia (44%), Africa (24%), and the Western Pacific (18%). Eight countries accounted for two-thirds of the global total, with India (27%), China (9%), and Indonesia (8%) being the top three countries on that list (2), Specifically, in Indonesia, there were 566,623 TB cases in 2018; 44% of the total cases were found in the most populated areas such as East Java, West Java, and Central Java (4).

Most TB patients are curable by a course of treatment, which is globally affordable, although this treatment takes a minimum of 6 months to complete and 2 years or longer for multidrug-resistant tuberculosis (MDR-TB) (5). The current TB control strategy is founded on early case detection and complete treatment to minimize transmission and prevent the emergence of drug resistance. However, even though many patients initiate TB treatment each year, they still face challenges complying with daily medication, causing many to adhere inconsistently or to stop prematurely. Treatment interruption increases the risk for acquired drug resistance, treatment failure, disease progression, relapse, and death; it also prolongs transmissibility (6).

In Indonesia, in particular, the incidence, and by consequence, the human and financial cost of TB infections remains extremely high (7). Some of the factors that cause loss to medical follow-up have been described in previous studies (8–11). Besides that, adherence to treatment completion is lower when patients have a negative treatment experience, e.g., when access to care involves substantial travel time, lost earnings, and other patient expenditures; when adverse drug reactions are frequent or consequential; or conversely, when patients feel better, and their motivation to finish treatment declines (12). Furthermore, in Indonesia, the role of community education has been described as low and impacting adversely on the effectiveness of TB treatment in the population, though these studies are still few and over short periods of time, for example, in Surakarta and Flores, and cannot necessarily be generalized over the entire Indonesian geographical and cultural complexity (13, 14).

Psychosocial support for Indonesian TB patients is a significant component in the management of TB drug side effects. Drugs supervisors (Pengawas Menelan Obat) are the people tasked to provide education and encouragement to the patients to maintain their treatment, organizing, when possible, individual meetings, community meetings, as well as support group meetings. The drug supervisors must be people who are already trained and accepted by the TB patients (15). The need for close, regular contact between caregivers and TB patients receiving treatment has been long recognized and remains topical (16). However, supervision by health workers is costly and requires a large number of health workers available in the field who are also appropriately trained. In the case of Indonesia, with a large population, dispersed over a geographically challenging terrain, this is not a realistic option. Therefore, novel technological solutions have to be deployed to enhance the national screening, treatment, and follow-up policies.

One of these novel technological solutions to TB treatment monitoring is telephone video communication, enabling health professionals to watch patients take their medication, address patients' concerns, and provide advice and support (17–19). Video (or virtually)-observed therapy (VOT) was piloted originally by using videophones connected to telephone landlines. This has now evolved toward using mobile telephones with video applications (smartphones) and even tablet computers. All these devices are becoming increasingly affordable and reliable in high- and low-income settings, such as Indonesia, while the coverage of cellular and internet networks is increasingly available in places where telephone landline services had never existed or are facing obsolescence.

This manuscript describes the outcome of a qualitative pilot study, based on a series of focus groups, to assess the knowledge—attitudes toward and behaviors on the potential utilization—of mobile technology for health purposes with a particular focus on TB treatment follow-up. Similar studies have taken place previously in other countries, though the global picture on the subject and, in particular, in the areas addressed by the current manuscript, remains incomplete (20–23). The focus groups were organized through the collaboration of UCL, the INDOHUN (Indonesia One Health University Network) consisting of 34 faculties from 20 universities across Indonesia (24) and Gadjah Mada University and took place in Yogyakarta, Indonesia, in the late 2018, and included various stakeholders [academic researchers, policy-makers, frontline healthcare staff from primary healthcare centers (Puskesmas), and patients] so that a deeper and more inclusive understanding can be pursued. Focus groups were not a mix of all the above stakeholders, but were focusing on one of the stakeholder groups at any one time. The four stakeholder groups were identified as critical, as such topics in Indonesia are still mostly within the academic sphere, with occasional pilot applications. At the same time, healthcare provision for the majority of the population is public through a recently introduced universal health coverage scheme (25). Therefore, policy-makers play a crucial role in the potential adoption process. Furthermore, the frontline staff and patients are providing a viewpoint from the potential implementation perspective, as evidence regarding optimal patient engagement is scarce globally (26). It was envisaged that in this way, top–down as well as bottom–up views will be captured.

This is the first time to our knowledge that this topic is discussed in this manner in Indonesia—outside of a narrow academic or political context and with a direct link to potential clinical utility, so that it can provide pilot data for the eventual development of a community-engaged intervention. Furthermore, the focus groups aimed to identify locally relevant priority areas where the participants felt that the implementation of mobile health technology would have the highest impact and reduce the burden of disease in the community.

## Methods

A community-based participatory research (CBPR) team comprising of active academic researchers in public health from the local university [Gadjah Mada University (UGM), Yogyakarta] and the INDOHUN network with projects active in community one-health engagement (Universitas Indonesia, Jakarta) sought to gain insight on person knowledge, attitudes, and behaviors related to mobile-technology utilization for TB treatment monitoring and follow-up, by conducting a qualitative research study. The UGM Institutional Review Board approved this study. The team jointly designed the study and selected a qualitative research approach using focus groups to address the specific aims. The CBPR team developed a focus group moderator's guide with semistructured questions related to the use of mobile health technology (the list of questions is available in Table 1).

**Table 1:** The list of questions used in all Focus Group Discussions following a short introduction on mobile-enabled health technologies for a potential implementation in TB remote observation and treatment monitoring in Indonesia.

1) Have you previously used or had experience with digital health technology?
2) What constraints do you experience when following the TB treatment process over a long time?
3) Is the monitoring from clinical staff needed during the long treatment process? Why is it needed?
4) Has the monitoring of treatment by clinical staff been done for the entire treatment process?
5) What kind of health technology have you known so far especially in monitoring treatment of TB for the patients?
6) If the health system in Indonesia implements technology as in the video (Video Directly Observed Therapy for TB), will you accept or refuse?
7) After hearing about the V-DOT in the video, do you think digital technology can help in monitoring the treatment process, especially the disease with a long duration treatment process?
8) Do you think that such technology is effective to be implemented in Indonesia; how and how would it be regulated?
9) What possible constraints that will arise if the technology is implemented in Indonesia?
10) Any suggestions given for application development and implementation?

### Participants' Recruitment

The INDOHUN and UGM designed the recruitment process with the UCL research team providing additional input. Purposive sampling was used and planned with the goal of collecting data until we reached saturation (27). Recruitment focused on the four key stakeholder communities: academic researchers, frontline clinical staff, policymakers, and patients (28). The community-engaged recruitment included flyers and word-of-mouth (snowball sampling) to the local University (UGM), the local Department of Health, community-based primary healthcare centers, and clinics. Eligibility criteria were individuals who self-identified as belonging to the four stakeholder categories and being ≥18 years old.

### Focus Group Procedures

Focus groups, arranged per stakeholder group, allowed for observations of nonverbal behaviors and interactions as well as group dynamics and to elicit shared cultural meanings (29). The focus group moderator guide, containing semistructured questions and probes, was adapted from a study conducted in Pakistan by academic and community members (30). In that case, the community members and leaders were interested in increasing health and wellness by using biomedical research to increase health awareness in the community. Bicultural and bilingual moderators and note-takers were recruited and trained to complete the data collection. The INDOHUN team members had prior expertise with conducting community-based qualitative research and had appropriate training. The training aims to ensure understanding of the purpose and objective and to maintain effective community-based participatory relationship (31). The groups were not separated by gender as it was thought from a cultural perspective that participants would be sharing in mixed-gender groups, as well as the use of such technology is generally gender agnostic; hence, a participant separation might create false assumptions.

### Focus Group Data Collection

We conducted four focus groups (FGs) using bilingual, bicultural moderators with participants from local communities from April to October 2018. Several study participants had low levels of English proficiency. To ensure that participants understood the aims and purpose of the study, a short description was provided as introduction in Indonesian. The semistructured questions were stated in both English and Indonesian, while most of the discussion took place in Indonesian. The participants received a light meal for their time. The structure of the focus group was repeated over the different events; it included two short keynote presentations providing (i) the definitions of mobile health and a few short examples of potential public health use from other resource-restricted settings globally and (ii) the framework for the regulation and implementation of mobile health solutions within Indonesia. The aim of these short presentations was to provide a common starting point, as well as to delineate the area of interest across the different focus groups.

Prior to starting the focus group discussions, facilitators (a) introduced themselves, (b) outlined the structure of the focus group, (c) instructed in the importance of confidentiality, and (d) collected selected sociodemographic information (sex, age, TB status, and professional occupation) through a brief survey.

Focus groups facilitated in Indonesian were transcribed verbatim and translated into English by the UGM and INDOHUN teams. Data were de-identified and unique IDs given to each participant and moderator. Participants were asked general questions pertaining to beliefs of health, which progressed to the specific list of questions (Table 1) leading the subsequent discussions. The moderators were familiar with the questioning route and experienced enough to know which questions were the most important and which could be dropped momentarily and return to those later. Following this method, the discussion had a better flow than being stifled by strictly following the order of questions. The focus groups were audio recorded. Audio files of the interviews were transcribed into English.

### Qualitative Analysis

Members from the research team independently conducted an initial review and coded transcripts. Preliminary thematic groups were generated and were linked to interview texts using traditional content analysis. Emerging themes were discussed and presented at team meetings. During these meetings, discordant codes were discussed until consensus was reached.

There are advantages and challenges in conducting qualitative research via focus groups. For example, focus groups, not individual interviews, were chosen as the preferred method because data are obtained from the communication between participants, as they share experiences and comment on different perspectives. This would be suppressed during a more formal interview setting. Also, sometimes, participants are more open when less inhibited members explore difficult topics and more open in a group format (32). The flow of the discussion was facilitated by using Indonesian throughout, so it is not stifled by any potential translation requirements. On the other hand, the process of analyzing the data was found to be time consuming. Factors such as the dynamics within the groups, between-group variations, and the need for translation, all contributed to the complexity of data and analysis.

### Validation of Findings

Uncertainties or disagreements were resolved by consensus discussion among the moderators and participants during the focus group. The identified themes are expected to be presented at an annual meeting of public health and research stakeholders by the INDOHUN colleagues (including some of the focus group participants) to confirm that interpretation of results was consistent with the intended meaning.

## Results

The CBPR team recruited 46 participants with a slight majority identifying as female (female 24, 52%; male 22, 48%). Sixteen participants were frontline clinical staff from primary healthcare centers (34%: 13 from the public sector, 3 from the private sector; FG1); 11 participants were academic researchers in public health (23%; FG2); 12 were public health practitioners (26%: 9 from public health offices of the local government, 3 from nongovernmental organizations; FG3), and 7 were ex-TB patients who have completed their respective treatments and are being followed-up actively (17%; FG4) (Table 2). The FGs were ~90 min in duration (range 80–110 min). FGs1–3 participants had completed tertiary education, FG4 participants had completed primary education.

**Table 2:** Summary of the participants' characteristics in the Focus Groups.

Total Number	46
Male/female	22(48%)/24(52%)
Front-line clinical staff	16 (34%)
Academic public health researchers	11 (23%)
Public health practitioners	12 (26%)
Ex TB-patients	7 (17%)

Three major themes emerged from the analysis of the data: (a) the increasing knowledge at all levels of healthcare activity in terms of the potential of mobile-enabled health technologies; (b) the structural and societal barriers associated with implementing potential mobile-based solutions; and (c) the need for government-driven, community-engaged implementations complemented by educational initiatives. There were no differences noted between communities or by gender, thus the results are consistent for the FGs.

### Theme 1: Increasing Understanding of Mobile Health Technologies Potential in Indonesia

Regarding overall knowledge, most FG participants agreed that there is a gradual implementation of mobile health technology in Indonesia through several applications known to them, which are approved by the Data Centre and Information Department of the Ministry of Health (Pusdatin, Kementerian Kesehatan). Such examples include the Integrated Referral Information System (Sistem Informasi Rujukan Terpadu, Sisrute) used in tertiary referral hospitals, the Integrated Nutrition Information System (Sistem Informasi Gizi Terpadu, SIGIZI Terpadu) used in primary healthcare centers (Puskesmas), the Integrated Emergency Response System (Sistem Penanggulangan Gawat Darurat Terpadu, SPGDT) used by the general public. Interestingly, while the professionals in the FGs were familiar at a minimum with the names of these systems and their basic aims, the patient FG was not fully aware of all of these systems or of their functions, though they were aware that some such systems exist. Specifically, both the patient and frontline staff FGs mentioned the current extensive use of automated messaging services and Whatsapp-based messaging as blanket reminder services for the adherence to the TB treatment between doctors' visits.

There was an overall positive attitude toward potential mobile health implementations. In particular, it was clear, especially by public health practitioners, that mobile health technology is considered vital in providing different solutions potentially to be implemented in almost every sector in Indonesian healthcare, from the general public, to frontline staff needs. However, from the outset of the discussions, several problems were identified relating to such a deployment. These can be summarized as lack of a planned integration between initiatives already taking place in different sectors. Therefore, it was considered critical that as part of the future deployment of mobile health technologies, the plans for their integration to existing healthcare functions (and complementarity with existing ones) should become a critical deciding factor.

Nomenclature and Health Literacy should also be mentioned here as a subtheme. From the FG discussions, participants mentioned that they lacked awareness of the scientific terms associated with mobile health. This lack of awareness hampers the understanding of the full potential of using such technologies and can create inconsistencies in terms of the expectations and influence a distant or less-less welcoming behavior toward mobile health. Across the different FGs, it was advised that if full-scale adoption by the public and the wider healthcare sector in Indonesia is the eventual aim, then the descriptive terms should exist in very simple language (in Bahasa Indonesia), accompanied by a number of explanatory pictures/depictions.

### Theme 2: Structural and Societal Barriers

Assessing the knowledge in terms of structural barriers, the lack of a specific legal framework was mentioned by healthcare professionals in the FGs. In Indonesia there exists a basic regulation and policies related to the application of technology [Law no. 19/2016, on Amendment to Law no. 11/2008 on Information and Electronic Transactions, Government Regulation (PP) No. 46/2014 on Health Information Systems]. Nevertheless, the latter law provisions do not specifically regulate the implementation of Mobile Health Technology. The Yogyakarta region specifically serves as a provincial model in Indonesia for community-oriented services utilizing communication technologies (“Jogja Cyber Province,” Governor Regulation No.42/2006). This might explain the heightened sense and general consensus observed in healthcare professionals, for the need of developing a legal framework to specifically address and regulate the implementation of mobile health technologies.

There was a general concern expressed as many FG participants spoke about several barriers to accessing healthcare. Such barriers contribute toward a more cautious attitude when it comes to the potential adoption on mobile health technology. For example, the language barrier was mentioned often, and in quite a similar manner, across the different groups, as language barriers can compromise the patient navigation. Participants mentioned they need someone who understands both the medical and technical languages and is able to translate the information “speak[ing] the dialect of the same community,” as frontline staff added. This allows the translation of information effectively from English into Indonesian and then into local, regional dialect(s). Numerous participants mentioned that the drugs supervisors (Pengawas Menelan Obat) might fulfill this role within the local context. Another possible barrier is the lack of confidence in western medical approaches, which needs to be taken into account when providing information about mobile health technologies originating from outside of Indonesia.

One constant subtheme across the different groups was the notion of “workload creep” as a barrier to eventual adoption of such technologies in the field. Participants felt people would tend to emphasize the potential benefit of mobile health technologies, without balancing appropriately potential pressures elsewhere in the system (from healthcare staff to administrative public health staff). For example, some participants mentioned that even when a video directly observed therapy for TB is implemented, while the potential patient benefits are clear, the potential workload implications on frontline healthcare staff are yet to be defined.

FG participants were also concerned about practical aspects: for example, the cost in accessing enough data quotes for video-based applications, and second, the quality of existing connections on mobile networks. While most of Indonesia is covered by mobile networks, and most Indonesians are owners of mobile phones, the quality of these networks is not uniform across all areas depending on population density and geography, nor is access to newer smartphones equally distributed in the population, allowing for video-based applications, which will affect the end-user behavior during a potential nationwide implementation of such initiatives, especially in remote and poorer areas of the country.

### Theme 3: Governmentally-Supported, Community-Driven Implementation, Complemented by Education

FG participants expressed a wide range of responses and opinions regarding the potential models of introduction and implementation of such mobile health technologies. The overall knowledge consensus was that an initiative, such as a potential one relating to TB treatment monitoring and follow-up, should be nationwide and supported by the central government, in line with other such recent initiatives [e.g., Antimicrobial Resistance Control National Action Plan (NAP/2016), Universal Health Coverage through the National Health System (Badan Penyelenggara Jaminan Sosial/2014), etc.].

Several examples participants mentioned were of implementation models driven by primary or tertiary healthcare structures. In the particular case of existing TB pathways in Indonesia, the current national healthcare system expects most TB patients to be treated by primary healthcare centers, with referrals to specialist tertiary centers be limited only to MDR-TB patients. Navigating through this tiered system is already challenging for individuals who are of the lowest income and/or educational levels, often resulting to a delay or interruption of patients' treatments. Additionally, there is limited data linkage/transfer between the different healthcare tiers; thus, any mobile-driven technology would need to bridge and converse with the different healthcare tiers, as well as be resilient to limitations in terms of healthcare data availability.

Therefore, in some FGs, the favorable position was that mobile healthcare technologies focusing on TB should be supported by the central government/public health departments through the central framework and overseeing mechanisms, and be driven in terms of implementation by the primary healthcare centers. This allows a close support of the initiative by drugs supervisors and an easy/ier information flow to patients. Across each FG, participants mentioned the importance of involving influential community leaders like civic leaders in helping to provide awareness of such a potential initiative.

The potential of such mobile health technologies for a number of overlapping healthcare aspects (e.g., TB and other infectious diseases) was currently not promoted in public health efforts. A number of FG participants stated that there is a paucity of pilot studies to support the introduction of such technologies. A participant in a frontline medical unit stated she did not know anything about mobile-driven technologies until she did her final year of training. When asked, “If there is an official approval process for allowing the introduction of such technologies?,” several members mentioned they did not know what such a process might be and needed more information. For instance, one participant mentioned, “[the] need for specific regulation to allow potential implementation.” There was also a lack of awareness of the breadth of such mobile-driven health technologies. One participant maintained that “there are different types of these applications” but “didn't have access to information about the different kinds.” However, participants were aware that they are not utilized for clinical purposes in Indonesia; some (six participants; 13%) have used applications for personal fitness/well-being requirements, and a very low number (three participants; 6%) for managerial purposes within their healthcare organization.

Continuing with the overall positive attitude toward mobile health, participants in all FGs expressed interest in learning more about mobile health technologies. For example, the need to learn about the implementation in other countries/communities of similar societal challenges, and not from more advanced nations, which is applicable only to upper-class levels of society and not the majority of the Indonesian population. Others discussed that partnerships for health education and promotion were most appropriate with academic institutions and community-based organizations, to pilot test such implementation attempts and provide the first evidence from within Indonesia before embarking on a nationwide initiative. Participants indicated the importance of having someone explain the nature of mobile health technologies (as was provided during the opening of the FG sessions). Last, several participants felt that it is important for individuals to start discussing such technologies within academic institutions so as to increase awareness of the potential to eventually save lives. More importantly, participants recognized the need for Indonesia to move forward with the rest of the world in this developing healthcare field.

## Discussion

To our knowledge, this is the first qualitative research study examining knowledge, attitudes, and behaviors toward mobile health technologies in Indonesia and, in particular, for the treatment and follow-up of TB patients. Findings from this pilot study provide insight on areas of research focus. Having a moderator team consisting of members with demonstrated community-based engagements, from the INDOHUN network, helped to increase trust and foster an understanding of shared community values. Information from this study provides evidence to support the creation, testing, and eventual development of professionally and culturally appropriate strategies in Indonesia to introduce mobile health technologies, eventually for a wider public use.

The findings illustrate that general knowledge of mobile health technologies, of their legal framework of operations, and of their exact potential within the healthcare system can be characterized as incomplete or poor. There is general knowledge that such technologies exist, but not a detailed operational exposure to them and their potential applications. There was a general positive attitude throughout the FGs toward mobile health technologies and their potential applications, especially as the benefits to the population and potential reach and immediacy of applying this technology was evident to participants from the outset. At the same time, there was also concern on the number of challenges that lay ahead for such technologies to be applied on a larger scale or even nationwide. These challenges can be at the individual level (accessibility to mobile phones and/or networks, language/comprehension challenges), to the local level (local healthcare facility capacity and integration to other ongoing tasks and activities) to the national level (improved national framework for mobile health technologies). Last, the behavior toward the mobile health technologies can be characterized as cautious and distant overall. There have been previous successful as well as unsuccessful attempts in Indonesia in rolling out nationwide healthcare initiatives, and as such, a general cautious behavior has ensued. However, this does not seem to be specific to mobile health technologies, but rather relating to the magnitude and operational complexity of such a task.

The novel findings of this study were: (A) the willingness of participants to learn about these technologies, as during the FGs there were repeated questions to the moderators and other participants to provide examples and online points of reference for further investigation. (B) The open and welcoming attitude of the participants toward receiving such information even within frontline community settings. This was perhaps surprising, as frontline staff are under constant pressure and often under resourced; hence, a discussion on a potential new initiatives might have not been welcome, expected to be greeted with heightened concern. However, there is clear acceptance that the benefits of mobile health technology are likely to outweigh potential disadvantages stemming from any complexity of implementation. (C) The willingness of participants to back a government-supported, primary healthcare-driven set of such initiatives. Various barriers to the implementation, operation, and eventual adoption, were described. In other parts of the world, studies have reported poor uptake of such technologies when the communication between various sections of the healthcare system was suboptimal, as well as within different units; this was supported from the opinions shared in our study (33, 34).

A previous work showed an agreement by healthcare providers and public health professionals that mobile health technologies can improve patient care, as this technology is perceived as able to reach most patients, when needed (35, 36). While this constitutes a benefit for the patient population, in general, the same ability can be seen as a potential barrier due to increased workload (34, 37, 38) and distributed workflow by frontline healthcare staff (39, 40). Our study has demonstrated general alignment with these previous findings. In addition, our study indicates that participants do not have a good understanding of the different types, or of the range, of existing mobile health technologies. Culturally, participants did not view the introduction of new technology as a serious barrier in itself, but had to be well-supported through community engagement.

Our results indicate that participants had some level of understanding of the various models of implementing such healthcare initiatives, based on recent nationwide campaigns within Indonesia. However, further research and education are needed to enhance the understanding of the potential barriers and facilitators before aiming to adopt such technologies widely. This was evident as several participants mentioned that access to reliable networks, data packages, and appropriate devices, would become immediate stumbling blocks in implementing such initiatives. This has been demonstrated previously in other resource-limited settings (41, 42).

Another subtheme emerging as a barrier is language and understanding. This notion is quite consistent with previous research in low-resource settings, and geographies where multiple languages coexist (43, 44). Ways that were suggested to empower and enable the community were to utilize community-based organizations, such as primary healthcare centers as a source of health education. Finally, it is worth mentioning a potential friction point, where there is a willingness to progress by adopting and embracing technological advances, while there still remains, in some part of the population, a mistrust of western medicine and medical approaches. The latter are likely to be entirely incorporated into mobile health technology solutions and, thus, would be interesting to observe how such beliefs can impact any potential adoption by the community.

## Limitations

The limitation of our study is that it might not be generalizable to the populations of South-East Asia, indeed, even Indonesia itself. Recruitment for the focus groups was limited by the sample size, as this was a pilot study with insufficient resources to conduct additional focus group sessions to allow for a more comprehensive comparison between groups (i.e., age, gender, education, etc.). Moreover, the focus group participants were self-selected and may not represent the majority of the population or the majority of opinion within their respective population groups. Another limitation is the need for having these observations validated through their presentation to the stakeholders as well as to the Indonesian healthcare community to ensure that the presented views are, indeed, widely representative. Furthermore, we acknowledge that the academic medical centers included in this study do not represent all types of facilities in which people are hospitalized and that any potential implementation might differ according to local needs and requirements. Nonetheless, we acknowledge the early nature of our findings in this rapidly evolving field. Finally, despite our efforts, our findings may not be exhaustive; they more than likely underscore considerable challenges and barriers to implementation and adoption as perceived by stakeholder participants.

## Conclusion

The entire issue of mobile health technologies, especially for remote patient monitoring, is attracting a great deal of attention worldwide because it presents a unique way to provide information and resources to healthcare professionals and patients alike, and may be a promising tool to support healthcare (45). The findings from the current pilot qualitative study focus on Indonesia due to the paucity of available data and the high healthcare need in terms of TB burden. The current work has identified challenges of implementation, integration, and health literacy that need to be addressed prior or during the future deployment of mobile health technologies. Additionally, it has identified the need for legal regulations and policies to allow for implementation and integration of mobile health technologies as well as their integration to existing healthcare functions. Further issues such as workload creep for frontline health workers and the literacy concerning both medical and technological languages were identified. Finally, there were concerns raised around the issue of accessibility of such technologies across the population, as well as governance and regulating of such technologies.

The themes expressed through the FGs provide a common ground, making it possible to better understand the challenges and opportunities related to mobile health technology utilization. While some of the aspects to the potential adoption are similar to those identified in systematic reviews about other technological applications (ease of use, access, etc.), this study has enabled us to identify factors that are specific to mobile health in one of the most populous nations of the world, Indonesia.

Based on these early findings, our most notable recommendation is that the findings described in the current manuscript would need to be further validated by a wider participation from the existing focus group stakeholder categories and expanded, through additional focus groups with other key stakeholders (e.g., health app developers, mobile industry representatives). This should allow the development of a core set of understanding regarding the knowledge, attitudes, and behaviors of Indonesians toward mobile health technologies and advance safe, coordinated, and effective dialogue and implementation of such technologies for patient-centered care. Further qualitative and quantitative research should build upon these concepts, enhance the themes we identified, test design assumptions, and measure the impact on outcomes.

## Disclaimer

Where authors are identified as personnel of the International Agency for Research on Cancer /WHO, the authors alone are responsible for the views expressed in this article, and they do not necessarily represent the decisions, policy, or views of the International Agency for Research on Cancer/WHO.

## Data Availability Statement

The datasets generated for this study are available on request to the corresponding author.

## Ethics Statement

The studies involving human participants were reviewed and approved by UGM Institutional Review Board. The patients/participants provided their written informed consent to participate in this study.

## Author Contributions

DA, ZK, and WTA contributed to study concept and design. WTA, RA, HD contributed on acquisition of data, data analysis, interpretation of data, and drafting of the manuscript. ZK, DN, WA, and AH contributed in interpretation of data, and critical revision of the manuscript and final approval of the manuscript. All authors contributed to manuscript revision, read, and approved the submitted version. All authors contributed to the article and approved the submitted version.

## Conflict of Interest

The authors declare that the research was conducted in the absence of any commercial or financial relationships that could be construed as a potential conflict of interest.
