# Supplementary material for: Knowledge, Attitudes, and Behaviors on Utilizing Mobile Health Technology for TB in Indonesia: A Qualitative Pilot Study
Source: Front Public Health. 2020 Oct 6;8:531514. doi: 10.3389/fpubh.2020.531514 (PMC7573209; doi:10.3389/fpubh.2020.531514)
Supplement: Supplementary file 1 [file Table_1.DOCX]

Table 1: The list of questions used in all Focus Group Discussions following a short introduction on mobile-enabled health technologies for a potential implementation in TB remote observation and treatment monitoring in Indonesia.

1. Have you previously used or had experience with digital health technology?
2. What constraints do you experience when following the TB treatment process over a long time?
3. Is the monitoring from clinical staff needed during the long treatment process? Why is it needed?
4. Has the monitoring of treatment by clinical staff been done for the entire treatment process?
5. What kind of health technology have you known so far especially in monitoring treatment of TB for the patients?
6. If the health system in Indonesia implements technology as in the video (Video Directly Observed Therapy for TB), will you accept or refuse?
7. After hearing about the V-DOT in the video, do you think digital technology can help in monitoring the treatment process, especially the disease with a long duration treatment process?
8. Do you think that such technology is effective to be implemented in Indonesia; how and how would it be regulated?
9. What possible constraints that will arise if the technology is implemented in Indonesia?
10. Any suggestions given for application development and implementation?
