# Supplementary material for: Knowledge, Attitudes, and Behaviors on Utilizing Mobile Health Technology for TB in Indonesia: A Qualitative Pilot Study
Source: Front Public Health. 2020 Oct 6;8:531514. doi: 10.3389/fpubh.2020.531514 (PMC7573209; doi:10.3389/fpubh.2020.531514)
Supplement: Supplementary file 2 [file Table_2.DOCX]

| Total Number | 46 |
| --- | --- |
| Male/Female | 22(48%)/24(52%) |
| Front-line clinical staff | 16 (34%) |
| Academic public health researchers | 11 (23%) |
| Public health practitioners | 12 (26%) |
| Ex TB-patients | 7 (17%) |

Table 2: Summary of the participants’ characteristics in the Focus Groups.
